# Unmasking hemoglobin Köln: a rare cause of discrepancies between pulse oximetry and arterial oxygen saturation—a case report

**DOI:** 10.3389/fmed.2024.1368068

**Published:** 2024-09-06

**Authors:** Awni Alshurafa, Ahmed Elsabagh, Koutaibah Rida Obaid, Youssef Elhaji, Mohamed A. Yassin

**Affiliations:** ^1^Hematology Department, Hamad Medical Corporation, Doha, Qatar; ^2^College of Medicine, Qatar University, Doha, Qatar; ^3^Internal Medicine Department, Hamad Medical Corporation, Doha, Qatar; ^4^Diagnostic Genomic Division, Department of Laboratory Medicine and Pathology, Hamad Medical Corporation, Doha, Qatar

**Keywords:** hemoglobin, hemolysis, unstable hemoglobin, hemoglobinopathies, hypoxia

## Abstract

Discrepancies between pulse oximetry and arterial oxygen saturation can pose challenges in clinical assessment. Possible underlying causes include poor peripheral perfusion, skin pigmentation, motion artifacts, and conditions like unstable hemoglobin and methemoglobinemia. Unstable hemoglobin variants, such as hemoglobin Köln, are rare inherited mutations affecting globin genes, potentially disrupting the folding, assembly, or interactions among subunits in globin molecules and the essential interactions between heme and globin for oxygen-binding properties. In this case report, we present the case of a 44-year-old Arabic woman who underwent extensive investigations due to disparities in pulse oximetry and arterial oxygen saturation, ultimately leading to the diagnosis of the unstable hemoglobin variant, hemoglobin Köln.

## Introduction

Unstable hemoglobin variants, caused by inherited variants affecting globin genes, are generally rare. Each variant, with few exceptions, is usually restricted to a single pedigree. These variants can impact alpha, beta, gamma, or delta globin chains and have the potential to interfere with the folding, assembly, or interactions among subunits in globin molecules, as well as interactions between heme and globin. The interaction between globin and heme is essential in shaping the oxygen-binding features and maintaining the stability and solubility of the molecule (1, 2).

Hemoglobin Köln is a rare genetic variant characterized by altered oxygen-binding properties. It occurs due to the substitution of valine with methionine at position β98 (FG5), leading to increased oxygen affinity and potential instability, likely attributed to heme depletion. It usually presents with mild anemia, reticulocytosis, and splenomegaly, comprising 10 to 25 percent of the total hemoglobin in heterozygotes. The unreliable readings of pulse oximetry in individuals with hemoglobin Köln and other unstable hemoglobin variants arise due to the partial lack of heme molecules in some tetramers (3, 4).

In this case report, we present a challenging case of a 44-year-old woman extensively investigated for disparities between Pulse Oximetry and Arterial Oxygen Saturation. Ultimately, an unstable hemoglobin variant, specifically hemoglobin Köln was diagnosed.

## Case report

A 44-year-old Algerian woman with a history of gallbladder stones presented to the emergency department with right upper quadrant pain. Upon initial assessment, she was vitally stable, not in apparent respiratory distress, and with normal blood pressure and heart rate. However, she was found to be hypoxic on room air with a pulse oximetry reading of 88% oxygen saturation. Physical examination was unremarkable except for right upper quadrant tenderness and guarding. History taking revealed no family history of hematological disorders. Her initial lab tests revealed hemoglobin of 13.7 gm/dL (normal >12 gm/dL), RBC of 4.6 × 10^6^/uL (normal 3.8–4.8 × 10^6^), white blood cells of 6.4 × 10^3^/uL (normal 3.5–10.5 × 10^3^), platelets of 212 × 10^3^/uL (normal 150–450 × 10^3^), mildly elevated bilirubin and normal renal and liver function tests (Table 1). As a workup for right upper quadrant pain, she underwent an abdominal ultrasound which revealed mild splenomegaly measuring 12.2 cm, gallbladder stones, and inflammation. Thus, she was admitted under acute care surgery for laparoscopic cholecystectomy.

**Table 1:** Patient initial laboratory results upon presentation.

Parameter	Value	Normal range
WBC	6.5 × 10^3^/uL	4.0–10.0
RBC	4.7 × 10^6^/uL	3.8–4.8
Hgb	13.7 gm/dL	12.0–15.0
Hct	%47.3	36.0–46.0
MCV	100.4 fL	83.0–101.0
MCH	29.1 pg	27.0–32.0
MCHC	29.0 gm/dL	31.5–34.5
Platelet	212 × 10^3^/uL	150–410

During her admission, she remained hypoxic with oxygen saturation ranging from 85 to 90% on room air. She was placed on supplemental oxygen via nasal cannula and face mask without improvement of her oxygen saturation reading on pulse oximetry. However, she did not complain of any respiratory symptoms and was comfortably breathing on room air. Upon further history taking, she mentioned she had a long-standing history of sudden, cold-independent bluish discoloration of her fingers and mild dyspnea on exertion for the past few years. A review of her medical records revealed that she had chronic low oxygen saturation readings incidentally found during routine medical checkups. Accordingly, she underwent extensive pulmonary and cardiac evaluations with pulmonary function tests, arterial blood gas, bronchoscopy with bronchoalveolar lavage, CT pulmonary angiography, echocardiography, and V/Q scan which all turned out to be unremarkable. Moreover, she was evaluated in a rheumatology clinic due to bluish discoloration of her fingers. Nevertheless, the results of all autoimmune work up (Myoglobin, Creatinine kinase, Cryoglobulin, ANCA, ANA, Anti CCP, C3, C4, rheumatoid factor, Ani-Jo1, Anti LA, Anti RO, and Anti RNP) came back negative.

She also mentioned getting frustrated by all the previously mentioned extensive investigations without reaching a clear diagnosis. Consequently, she was discharged from rheumatology and pulmonology clinics. Upon reviewing her previous blood tests, it was noted that her hemoglobin was ranging from 10 gm/dL to 13 gm/dL (normal >12 gm/dL). As a result, an anemia workup revealed a normal iron profile, thyroid function, vitamin b12, and folate but peculiar hemoglobin electrophoresis results. The result of hemoglobin electrophoresis revealed 85.5% HgbA, 3% Hgb A2, 1% Hgb F and 10.5% of others. Electrophoresis was interpreted with the following comment (presence of an abnormal peak of 10.6% with a retention time of 4.88 min which could not be separated from HbA by manual electrophoresis. The high-performance liquid chromatography pattern is close to that of Hb Koln which is a beta chain variant, however, molecular analysis is required for identification). Peripheral smear showed mild normochromic normocytic anemia without Heinz bodies. Urinalysis was only remarkable for trace blood for which urine microscopy was sent for confirmation and revealed no WBC and 5 RBCs (normal 0–9).

Arterial blood gas revealed SO2 of 97% (normal 95–99%), O2Hb of 93.7% (normal: 94–98%), COHb of 1.3% (normal 0.5–1.5%), with PH of 7.49 (normal 7.35–7.45), PaO2 of 96 mmHg (normal 83–106 mmHg) and PaCO2 of 33 mmHg (normal 35–45 mmHg) (Table 2). The G6PD screening test came back negative. Laboratory results findings were consistent with a beta chain variant hemoglobin Köln (Hb Köln) which explains the discordance between pulse oximetry reading and PaO2 on arterial blood gas. She was subsequently cleared for surgery and underwent laparoscopic cholecystectomy without complications. Surgery was done under general anesthesia and she was continuously being monitored by pulse oximetry which showed stable oxygen saturation around 90% on 21% FiO2. Molecular genetic testing was requested for confirmation.

**Table 2:** Patient’s initial arterial blood gas results on room air.

ABG	Value	Normal range
pH Art-POC	7.493	7.35–7.45
PO2 Art-POC	96 mmHg	83–108
PCO2 Art-POC	33 mmHg	35–45
BG Lac Art-POC	0.50 mmol/L	0.36–1.6
tHb Art-POC	11.1 gm/dL	12–16
SO2 Art-POC	97.0%	95.0–99.0
O2Hb Art-POC	93.7%	94.0–98.0
COHb Art-POC	1.3%	0.5–1.5
MetHb Art-POC	2.1%	0.0–1.5
HCO3 Art-POC	25.6 mmol/L	23.0–29.0

Hemoglobin beta gene (HBB) sequencing revealed Heterozygosity for HBB:c.295G > A [Hb Köln, also known as Hb San Francisco (Pacific)] and heterozygosity for HBB:c.-106G > C, a variant of uncertain significance. Heterozygots for the c.-106G > C variant have been reported with normal hematological and electrophoretic features (HbVar ID 2601). Thus, the patient was diagnosed with the rare beta chain variant Hb Köln, and is currently being followed by the hematology clinic.

## Discussion

Discrepancies between pulse oximetry (SpO2) and arterial oxygen saturation (SaO2) can pose challenges in clinical assessment. Pulse oximetry estimates oxygen saturation by shining light through the skin, but several factors can lead to differences between SpO2 and SaO2. These factors include poor peripheral perfusion, skin pigmentation, motion artifacts, and blood-related issues such as hemoglobinopathies, methemoglobinemia, and carboxyhemoglobinemia. Hemoglobinopathies may lead to fluctuations in SpO2 and SaO2 by affecting oxygen-binding characteristics, influencing light absorption, and introducing unstable hemoglobin variants. Among the over 1,200 described hemoglobin variants, approximately 150 are classified as unstable alpha globin or beta globin variants (5–7).

Hemoglobin Köln is a rare variant caused by a genetic variant in the beta-globin gene. Hb Köln is found in various racial and ethnic groups and is often observed as a *de novo* variant (HbVar ID 448). Unlike normal hemoglobin, it exhibits a high affinity for oxygen without requiring compensatory erythrocytosis and demonstrates easy heme dissociation.

Individuals with hemoglobin Köln may typically exhibit Heinz body hemolytic anemia (8). However, in this reported case, the absence of Heinz bodies in the red cells could be attributed to the active functioning of the patient’s spleen. Hemoglobin Köln is caused by a substitution of adenine (A) for guanine (G) at position c.295 of the HBB gene leading to the replacement of the amino acid valine at position 98 with a methionine. The partial lack of heme molecules in some of the tetramers of hemoglobin Köln makes the use of pulse oximetry unreliable in these individuals (9, 10). Methemoglobinemia can coexist with an unstable hemoglobin variant, such as hemoglobin Köln, leading to heightened discrepancies in oxygen saturation. The structural anomalies of hemoglobin Köln, coupled with the diminished oxygen-carrying capacity of methemoglobin, contribute to significant inaccuracies in pulse oximetry readings. This complex interplay highlights the intricate interactions among hemoglobin variants and related conditions, affecting the accuracy of assessments for oxygen transport (11).

Perioperative management becomes particularly challenging in cases involving unstable hemoglobin like Hemoglobin Köln, where the pulse oximetry may not reflect the actual body oxygenation. Given the potential discrepancies in oxygen saturation readings, utilizing alternative methods to monitor oxygenation, such as arterial blood gas analysis, becomes essential for a more precise assessment. Implementing precautionary measures, including maintaining adequate hydration and avoiding hemolysis-inducing triggers, with continuous vital signs monitoring becomes crucial. A collaborative effort between anesthesiologists and hematologists is strongly recommended to ensure a thorough and tailored approach to the perioperative period for individuals with unstable hemoglobin (12, 13). In our patient, there was a significant challenge in reaching the diagnosis. A simple test like hemoglobin electrophoresis with meticulous interpretation could have avoided the use of expensive and extensive investigations leading to patient dissatisfaction. Furthermore, awareness of the existence of such hemoglobin variants and their effect on pulse oximetry reading is crucial for pre-anesthesia assessment to avoid any delay or cancelation of high-priority surgeries. To the best of our knowledge, this is the first reported case of Hemoglobin Köln in the Arab population.

In conclusion, when SpO2 readings appear inconsistent with the patient’s clinical condition, the diagnosis of unstable hemoglobin variants should be considered, especially in cases of oxygen desaturation, with normal respiratory and cardiac investigations. Understanding their impact on oxygen saturation measurements is crucial for providing accurate diagnostics, tailored treatment strategies, and preventing unnecessary investigation.

## Data availability statement

The raw data supporting the conclusions of this article will be made available by the authors, without undue reservation.

## Ethics statement

The studies involving humans were approved by Medical Research Council at Hamad Medical Corporation. The studies were conducted in accordance with the local legislation and institutional requirements. Written informed consent for participation was not required from the participants or the participants' legal guardians/next of kin in accordance with the national legislation and institutional requirements. Written informed consent was obtained from the individual(s) for the publication of any potentially identifiable images or data included in this article.

## Author contributions

AA: Conceptualization, Writing – original draft, Writing – review & editing. AE: Funding acquisition, Writing – original draft, Writing – review & editing. KO: Data curation, Writing – original draft. YE: Conceptualization, Writing – review & editing. MY: Funding acquisition, Supervision, Writing – review & editing.
